# Effects of Dynamic Hyperinflation on Left Ventricular Diastolic Function in Healthy Subjects — A Randomized Controlled Crossover Trial

**DOI:** 10.3389/fmed.2021.659108

**Published:** 2021-05-04

**Authors:** Matthias Helmut Urban, Anna Katharina Mayr, Ingrid Schmidt, Erwin Grasmuk-Siegl, Otto Chris Burghuber, Georg-Christian Funk

**Affiliations:** ^1^Department of Internal and Respiratory Medicine, Klinik Floridsdorf, Vienna, Austria; ^2^Karl-Landsteiner-Institute for Lung Research and Pulmonary Oncology, Vienna, Austria; ^3^Otto Wagner Hospital, Ludwig-Boltzmann Institute for Lung Health, Vienna, Austria; ^4^Institute for Physical and Rehabilitation Medicine/Cardiorespiratory Therapy, Klinik Floridsdorf, Vienna, Austria; ^5^Medical School, Sigmund Freud University, Vienna, Austria; ^6^Department of Internal and Respiratory Medicine, Klinik Ottakring, Vienna, Austria

**Keywords:** heart failure, diastolic dysfunction, diastolic filling, dynamic hyperinflation, positive end-expiratory pressure, chronic obstructive pulmonary disease

## Abstract

**Objective:** Diastolic dysfunction of the left ventricle is common in patients with chronic obstructive pulmonary disease (COPD). Dynamic hyperinflation has been suggested as a key determinant of reduced diastolic function in COPD. We aimed to investigate the effects of induced dynamic hyperinflation on left ventricular diastolic function in healthy subjects to exclude other confounding mechanisms associated with COPD.

**Design:** In this randomized controlled crossover trial (NCT03500822, https://www.clinicaltrials.gov/), we induced dynamic hyperinflation using the validated method of expiratory resistance breathing (ERB), which combines tachypnea with expiratory resistance, and compared the results to those of tachypnea alone. Healthy male subjects (*n* = 14) were randomly assigned to the ERB or control group with subsequent crossover. Mild, moderate, and severe hyperinflation (i.e., ERB1, ERB2, ERB3) were confirmed by intrinsic positive end-expiratory pressure (PEEP_i_) using an esophageal balloon catheter. The effects on diastolic function of the left ventricle were measured by transthoracic echocardiographic assessment of the heart rate-adjusted transmitral E/A-ratio and E/e'-ratio.

**Results:** We randomly assigned seven participants to the ERB group and seven to the control group (age 26 [24-26] vs. 24 [24-34], *p* = 0.81). Severe hyperinflation decreased the E/A-ratio compared to the control condition (1.63 [1.49–1.77] vs. 1.85 [0.95–2.75], *p* = 0.039), and moderate and severe ERB significantly increased the septal E/e'-ratio. No changes in diastolic function were found during mild hyperinflation. PEEPi levels during ERB were inversely correlated with the E/A ratio (regression coefficient = −0.007, *p* = 0.001).

**Conclusions:** Our data indicate dynamic hyperinflation as a determinant of left ventricular diastolic dysfunction in healthy subjects. Therapeutic reduction of hyperinflation might be a treatable trait to improve diastolic function in patients with COPD.

## Introduction

Chronic obstructive pulmonary disease (COPD) is among the most common causes of death worldwide (1). Despite decreasing mortality rates in some Western countries, the global mortality attributable to COPD has continued to rise over the last decades (2). Cardiovascular comorbidities are among the most relevant drivers of mortality in patients with COPD (3, 4).

Diastolic dysfunction of the left ventricle, along with systolic heart failure, coronary artery disease, and hypertension, has emerged as one of the most frequent comorbidities in COPD (5–7). The prevalence of diastolic dysfunction is variable and can reach as high as 90%, independent of disease severity, in COPD (8, 9). Patients with diastolic dysfunction are much more likely to have airflow limitation than patients with systolic dysfunction (10).

The current knowledge suggests that dynamic hyperinflation is a leading risk factor for diastolic dysfunction in COPD. Watz et al. (11) reported that the association of hyperinflation with left ventricular diastolic dysfunction was stronger than that of airflow limitation or diffusion capacity. These findings are confirmed by longitudinal data indicating that increasing hyperinflation is a major determinant of diastolic impairment of the left ventricle in COPD (12). A reduction in preload was subsequently suggested as a link between hyperinflation and reduced diastolic filling (11, 13). However, the pathophysiologic interaction between COPD and compromised diastolic function is complex, and there are numerous possible mechanisms (14, 15). In addition to hyperinflation, hypoxic vasoconstriction (9), arterial stiffness (16), pulmonary hypertension (17), ventricular interdependence (7), and subendocardial ischemia (18) have been introduced as potential links in this cardiorespiratory interaction. Hence, the induction of hyperinflation in healthy subjects is used to study the effects of intrathoracic pressure changes on the heart as an isolated mechanism (19). However, no study has investigated diastolic function during the induction of dynamic hyperinflation in healthy subjects.

We aimed to induce dynamic hyperinflation in subjects without COPD to study its effects on echocardiographically assessed parameters of diastolic function. We hypothesized that induced hyperinflation, indicated by positive end-expiratory pressure (PEEPi), would significantly reduce the E/A ratio and increase the E/e' ratio in subjects without cardiorespiratory diseases. Rejection of the null hypothesis would suggest that hyperinflation is a relevant mechanism for and a therapeutic target against diastolic dysfunction in patients with COPD.

## Materials and Methods

### Participants

This study included healthy subjects younger than 40 years of age recruited from the Medical University of Vienna. Subjects with obesity (i.e., body mass index ≥28), current or former cigarette smoking or respiratory disorders were excluded. Airflow limitation was ruled out via spirometry (Easyone, ndd medical technologies, Zurich, Switzerland). Further, exclusion criteria comprised arterial hypertension or overt cardiovascular diseases, including structural heart diseases. Finally, subjects with a baseline E/A ratio below 1.0 were excluded before randomization. After receiving detailed instructions about study-related procedures, each participant had to give written informed consent.

### Study Design and Intervention

This randomized controlled investigator-blinded crossover trial was conducted at the Department of Respiratory and Critical Care Medicine at the Otto Wagner Hospital, Vienna. Participants were randomly assigned to start with ERB or tachypnea. Randomization was conducted in blocks of four participants via an online program (www.randomization.com). Dynamic hyperinflation in the study group was induced by means of the recently validated expiratory resistance breathing (ERB) method (20). Briefly, hyperinflation was induced *via* airflow limitation by means of an expiratory airway stenosis. Inspiration remained unmodified due to a one-way valve coupled via a T-connector. A metronome was used in order to standardize participants' breathing pattern (i.e., respiratory rate and ratio of inspiration to expiration). Titration of hyperinflation was achieved *via* a stepwise reduction of the expiratory stenosis diameter (i.e., mild: 3.0 mm, moderate: 2.0 mm and severe: 1.5 mm). Standardized tachypnea, paced with a metronome (21), was used as the comparator intervention. After the first interventional phase (i.e., either ERB or tachypnea), the participants crossed over to the alternative intervention.

### Measurements

Diastolic function was assessed *via* echocardiography with the apical four-chamber view (Vivid S9, GE Healthcare, Fairfield, USA), following current recommendations (22), and was subsequently analyzed by a blinded investigator. The velocity of transmitral flow during early and late diastole was measured to quantify the E-wave, A-wave, and E/A ratio. The E/A ratio was corrected for a heart rate of 60/min, as tachycardia can cause fusion of the E- and A-waves with a false-positive diastolic filling pattern. Hence, we excluded all measurements where the A- and E-waves met at a velocity of at least 0.2 m/s. Instead, we calculated the maximum A-velocity and the heart rate-corrected E/A-ratio, which resulted in a more conservative reduction of the E/A-ratio. Tissue Doppler imaging of the septal and lateral e'-waves was used to quantify the E/e'-ratio. Echo parameters were assessed during expiration, and the mean value was derived from three cardiac cycles.

Prior to the study intervention, an esophageal balloon catheter (Model C76050U, Marquat Génie Biomédical, Boissy-Saint-Léger, France) was inserted according to current standards (23). *Via* a a pressure transducer, esophageal pressure was recorded by a validated pressure box (ICU-Lab, Kleistek Engineering, Bari, Italy). PEEPi was quantified by the negative deflection of esophageal pressure between the onset of inspiratory effort and the beginning of inspiratory flow (24).

### Statistics and Ethics

Data are presented as the means and 95% confidence intervals or as medians and 1st−3rd quartiles. Normal distribution was assessed by normal plots and were logarithm transformed if required. For intergroup comparison of baseline data, we used the Mann-Whitney *U*-test. Due to repeated observations, we used a generalized estimation equation with a normal probability distribution and an exchangeable correlation structure for between-group comparisons of echocardiographic and respiratory variables. Statistical analysis was calculated by STATA, version 14.2 (StataCorp, Texas 77845 USA).

Participants who discontinued the intervention prior to crossover were re-enrolled. Disruption after crossover resulted in exclusion from the study. The association between PEEPi and the E/A ratio was assessed by a generalized estimation equation with a normal probability distribution, an exchangeable correlation structure, and robust standard errors. To rule out potential confounding by randomization, we added a randomization group to the model.

The sample size was calculated by means of the primary outcome (i.e., E/A ratio) as a continuous variable from matched pairs during spontaneous breathing and ERB. In a pilot study, we found the effect size of matched pairs to be normally distributed with a standard deviation of 0.15. With an effect size of 0.17, we calculated a sample size of 10 participants to reject the null hypothesis with a probability of 0.9. The level of significance (i.e., type-1 error) was set at 0.05. The number of dropouts was estimated at four, yielding a final sample size of 14 participants. These calculations were made using PS – power and sample size (free software, version 3.0.43).

This trial was approved by the local Ethics Committee/Institutional Review Board (EK 15-209-1015). All study-related procedures were conducted in accordance with the Declaration of Helsinki. The study was registered with http://www.clinicaltrials.gov under NCT03500822 and is reported in accordance with the CONSORT statement (25).

## Results

### Subject Characteristics

**Figure 1:**
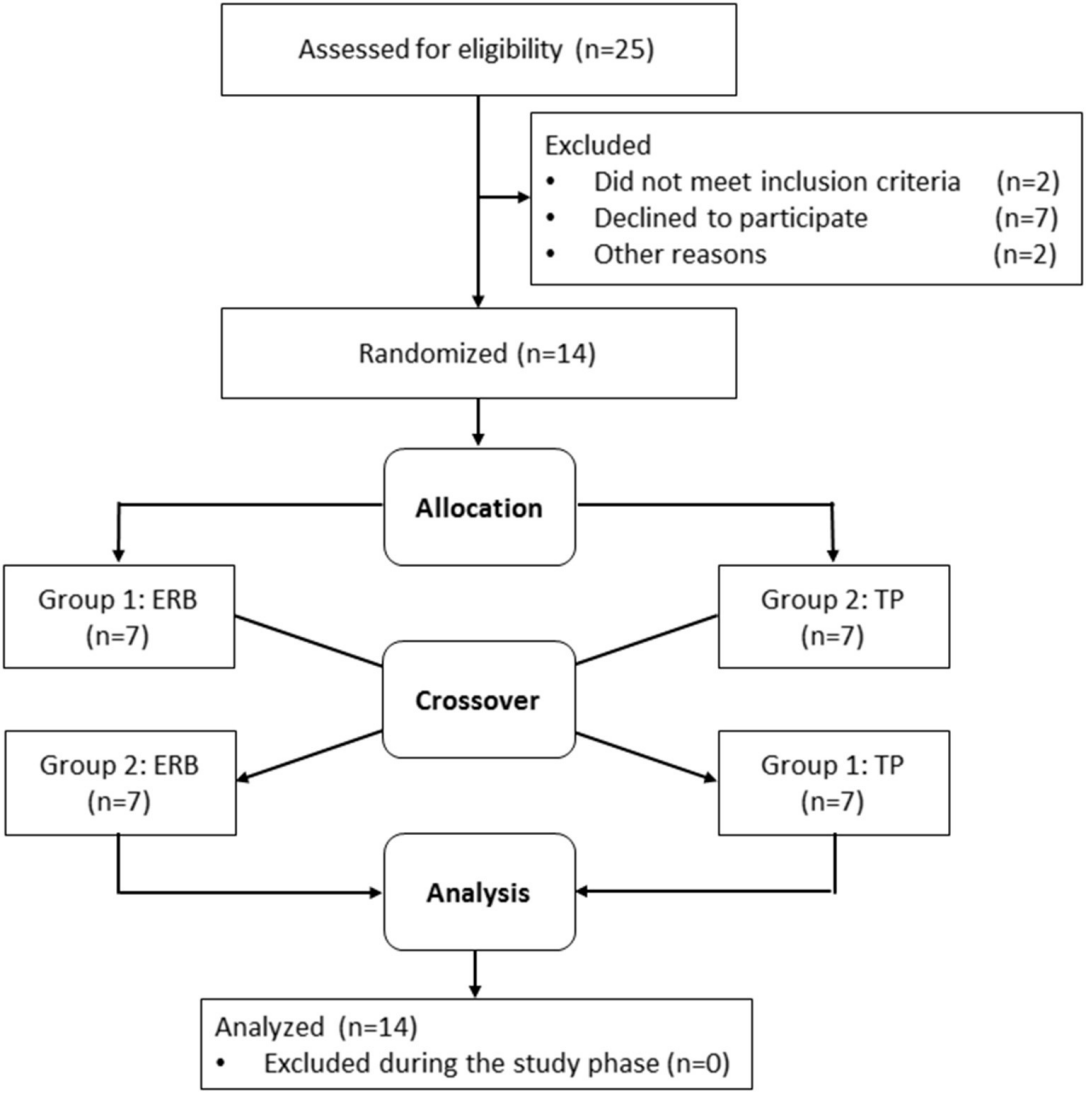
Participant flow chart and randomization. ERB, expiratory resistance breathing; TP, tachypnea.

Recruitment started in July 2017, and data collection was completed in November 2017. Twenty-five subjects were assessed for eligibility, and 11 subjects did not meet the inclusion criteria or declined to participate. None of the participants had to be excluded during the study, and seven subjects per study group were available for inclusion in the analysis. Participant flow is depicted in Figure 1. Mean age, height and weight were equally distributed between the two randomization groups. Baseline echocardiography showed no structural or functional abnormalities and no significant differences between the groups. Detailed subject characteristics are presented in Table 1.

**Table 1:** Baseline characteristics of study participants starting with expiratory resistance breathing (ERB, group 1) and tachypnea (TP, group 2).

	**Group 1 – ERB (*n* = 7)**	**Group 1 – TP (*n* = 7)**	***p*-value**
**Anthropometrics**			
Age, years	26 (24–26)	24 (24–34)	0.805
Height, cm	184 (179–187)	178 (177–184)	0.128
Weight, kg	77 (75–85)	76 (75–86)	1.000
BMI	23.7 (22.1–25.1)	24 (21.4–27.1)	0.456
BSA, m^2^	1.95 (1.93–2.08)	2.00 (1.92–2.09)	1.000
**Lung function testing**			
FEV1, L	4.96 (4.55–5.23)	4.55 (3.57–5.05)	0.259
FVC, % predicted	5.78 (4.97–6.43)	5.60 (4.66–5.73)	0.259
FEV1/FVC ratio	0.86 (82–0.89)	0.81 (0.77–0.88)	0.318
IC, L	3.68 (3.46–3.90)	3.62 (3.21–4.02)	0.670
MEF25, L/s	2.57 (1.95–2.65)	1.89 (1.53–2.53)	0.730
MEF50, L/s	6.25 (6.14–6.36)	5.66 (3.88–6.10)	0.290
MEF75, L/s	9.28 (8.38–10.24)	8.99 (7.01–9.95)	0.560
PEF, L/s	11.58 (10.93–12.45)	10.77 (10.20–10.78)	0.063
**Cardiovascular parameters**			
Heart rate, bpm	70 (62–71)	71 (66–78)	0.620
EF, percent	60 (57–64)	59 (56–63)	0.731
E/A ratio	1.59 (1.43–2.28)	1.80 (1.27–2.20)	0.710

*Data are presented as medians with 1st−3rd quartiles and were compared by a Mann-Whitney U-test. ERB, expiratory resistance breathing; TP, tachypnea; BMI, body mass index; BSA, body surface area; FEV1, forced expiratory volume in1 s; FVC, forced vital capacity; IC, inspiratory capacity; EF, ejection fraction; E/A ratio, ratio of E-wave to A-wave*.

### Cardiovascular Outcomes During Dynamic Hyperinflation

**Figure 2:**
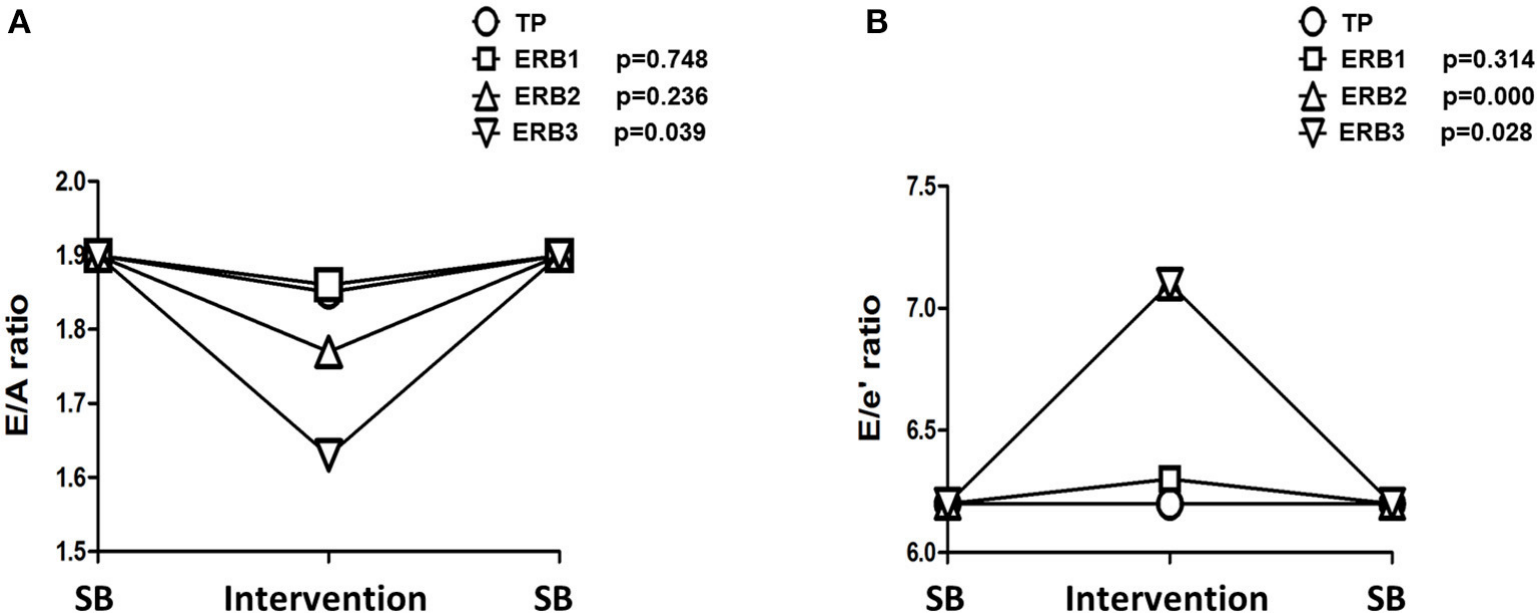
Line chart of changes in E/A **(A)** and E/e' **(B)** during tachypnea and expiratory resistance breathing with diameters of 3 mm (ERB1), 2 mm (ERB2), and 1.5 mm (ERB3). TP, tachypnea; ERB, expiratory resistance breathing; SB, spontaneous breathing.

Hyperinflation, expressed as PEEPi, showed a non-parametric distribution. Log transformed PEEPi revealed stepwise increases from tachypnea to ERB1, ERB2, and ERB3 (delta PEEPi: 5.27 [4.96–17.13], *p* < 0.006; 21.33 [11.82–25.34], *p* = 0.002; 36.27 [26.13–43.58], *p* = 0.009). PEEPi correlated inversely with inspiratory capacity (regression coefficient = −1.04; *p* < 0.0001). The relationship between the two is displayed in Supplementary Figure 1. During hyperinflation, we observed a significant increase in heart rate. The E/A ratio was significantly reduced from tachypnea to ERB1 and ERB3. The reduction in the E/A ratio was predominantly maintained by increases in the A-wave, whereas the E-wave remained unchanged by hyperinflation. Even after correction for a heart rate of 60/min, the E/A ratio was significantly reduced during ERB2 and ERB3. Tissue Doppler showed significantly elevated septal and lateral E/e' ratios during hyperinflation. The changes in E/A and E/e' during hyperinflation are depicted in Figure 2. All echocardiographic parameters during spontaneous breathing, tachypnea and hyperinflation are listed in Table 2. No adverse events were observed during this study.

**Table 2:** Cardiovascular outcome measures during spontaneous breathing (SB), tachypnea (TP) and expiratory resistance breathing with diameters of 3 mm (ERB1), 2 mm (ERB2), and 1.5 mm (ERB3).

	**SB**	**TP**	**ERB1**	***p*-value (vs. TP)**	**ERB2**	***p*-value (vs. TP)**	**ERB3**	***p*-value (vs. TP)**
Heart rate, bpm	67 (63–71)	71 (65–76)	74 (69–80)	0.357	76 (71–81)	0.134	80 (72–87)	0.036
MV Emax, m/s	0.82 (0.76–0.90)	0.80 (0.72 to 0.89)	0.82 (0.73–0.90)	0.254	0.84 (0.77–0.91)	0.080	0.81 (0.74–0.88)	0.512
MV Amax, m/s	0.50 (0.47–0.54)	0.54 (0.48–0.61)	0.57 (0.47–0.66)	0.285	0.63 (0.54–0.72	0.010	0.68 (0.59–0.77)	<0.0001
MV Amax^*^, m/s	0.50 (0.46–0.54)	0.51 (0.46–0.57)	0.49 (0.45–0.53)	0.319	0.57 (0.50–0.64)	0.002	0.62 (0.55–0.70)	<0.0001
MV E/A	1.71 (1.49–1.92)	1.58 (0.76–2.40)	1.55 (1.31–1.80)	0.767	1.43 (1.21–1.65)	0.044	1.26 (1.10–1.43)	<0.0001
MV_E/A^*^	1.72 (1.50–1.93)	1.56 (0.76–2.36)	1.71 (1.49–1.93)	0.973	1.52 (1.30–1.75)	0.009	1.34 (1.16–1.52)	<0.0001
MV E/A^#^	1.90 (1.66–2.14)	1.85 (0.95–2.75)	1.86 (1.63–2.10)	0.748	1.77 (1.59–1.96)	0.236	1.63 (1.49–1.77)	0.039
MV_E/A^*^^, #^	1.90 (1.66–2.14)	1.89 (0.93–2.85)	2.01 (1.76–2.26)	0.073	1.84 (1.64–2.05)	0.492	1.69 (1.50–1.87)	0.073
e' sept	0.14 (0.12–0.15)	0.13 (0.12–0.15)	0.14 (0.12–0.15)	0.799	0.12 (0.11–0.14)	0.052	0.12 (0.11–0.13)	0.075
E/e' sept	6.2 (5.7–6.8)	6.2 (5.6–6.7)	6.3 (5.7–7.0)	0.314	7.1 (6.4–7.8)	0.000	7.1 (6.2–8.0)	0.028
e' lat	0.17 (0.16–0.18)	0.16 (0.15–0.17)	0.16 (0.08–0.24)	0.498	0.15 (0.14–0.17)	0.655	0.15 (0.14–0.17)	0.212
E/e' lat	5.0 (4.4–5.5)	5.1 (4.5–5.8)	5.4 (4.8–6.0)	0.123	5.7 (5.0–6.3)	0.192	5.4 (4.5–6.3)	0.436

*Data are presented as mean values with 95% confidence intervals. The respective measures during spontaneous breathing periods prior to interventional periods are summarized as one mean value.SB, spontaneous breathing; TP, tachypnea; ERB, expiratory resistance breathing; MV_Emax, maximum early-diastolic transmitral flow; MV_Amax, maximum late-diastolic transmitral flow; MV_E/A, ratio of E-wave to A-wave; e' sept, early-diastolic tissue Doppler velocity at the septal mitral annulus; e' lat, early-diastolic tissue Doppler velocity at the lateral mitral annulus; E/e' sept, ratio of E to e' sept; E/e' lat, ratio of E to e' lat*.

* *non-fused;*

# *corrected for a heart rate of 60/min*.

### Association of Hyperinflation and Diastolic Function

**Figure 3:**
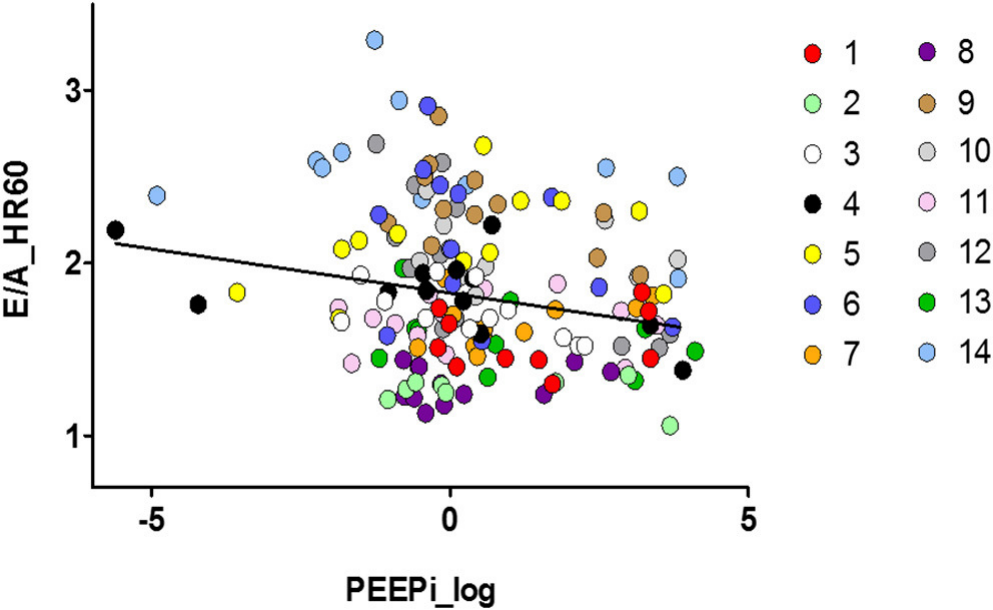
Scatter plot of the association between left ventricular diastolic function and dynamic hyperinflation of the lungs. Individual observations (1–14) are color coded. E/A_HR60, ratio of transmitral E- to A-wave corrected for a heart rate of 60/min; PEEPi log, log transformed intrinsic positive end-expiratory pressure (cmH2O).

The association between hyperinflation and the E/A ratio is illustrated in Figure 3. PEEPi showed a significant inverse correlation with the E/A ratio corrected for a heart rate of 60/min. As shown in Table 3, an increase of 1 cmH2O in PEEPi resulted in a reduction in the heart rate-corrected E/A ratio of 0.007. This association was independent of the randomization group (*p* = 0.936).

**Table 3:** Association between the E/A ratio and dynamic hyperinflation, adjusted for randomization.

	**Coefficient**	***p*-value**	**Lower 95% CI**	**Upper 95% CI**
PEEPi dyn, cmH20	−0.007	0.001	−0.011	−0.003
Randomization	−0.015	0.936	−0.391	0.360
Constant	1.903	<0.0001	1.245	2.561

*Association between the diastolic function of the left ventricle, expressed as E/A ratio and corrected for heart rate, as the constant variable and dynamic hyperinflation, expressed as intrinsic positive end-expiratory pressure; E/A ratio, ratio of maximum early- to late-diastolic flow at the mitral valve; lower 95% CI, lower 95% confidence interval; upper 95% CI, upper 95% confidence interval; PEEPidyn, dynamic changes in intrinsic positive end-expiratory pressure*.

## Discussion

In this randomized controlled trial, we demonstrate that dynamic pulmonary hyperinflation causes diastolic dysfunction in healthy individuals. The induction of hyperinflation caused a significant impairment of diastolic function, expressed as the E/A ratio, and an increase in ventricular filling pressures, expressed as the E/e' ratio. The amount of hyperinflation, as measured by PEEPi, was inversely associated with the E/A ratio. These findings indicate that diastolic dysfunction is determined by intrathoracic pressures and strengthen the role of hyperinflation as a therapeutic target for improving left ventricular diastolic filling and function in COPD.

The observed impairment of ventricular function and filling by induced hyperinflation is in concordance with previous studies in the field. Cheyne and colleagues (19) simulated COPD-specific hyperinflation in healthy subjects and found that left ventricular filling, quantified by end-diastolic volume, was significantly reduced following resistive loading of the respiratory system. Similarly, clinical observations of intubated patients with respiratory failure revealed reduced left ventricular end-diastolic volume and venous return *via* increasing extrinsic PEEP levels (26). Our data extend this knowledge by providing a detailed description of the early- and late-diastolic transmitral flow and E/e' ratio during dynamic hyperinflation. The amount of dynamic hyperinflation during ERB is comparable to the values observed in patients with COPD (20). COPD patients at rest revealed modest PEEPi levels ranging from 2.4 to 4.8 cmH2O (27, 28) which evidentially increase *via* voluntary tachypnea. During exercise, PEEPi was found to rise up to over 12 cmH2O (29), which is comparable to the 11.2 cmH2O during ERB with 3 mm stenosis diameter. Finally, during acute ventilatory failure, previous studies showed PEEPi levels of up to 20 cmH2O (30), which is in line with the 22.3 cmH2O of PEEPi measured during ERB with a stenosis diameter of 2 mm (20). Static hyperinflation and severe pulmonary emphysema have been identified as determinants of reduced end-diastolic volume by magnetic resonance imaging (31, 32). In the present study, we identified a significant negative association between the E/A ratio and dynamic hyperinflation, expressed *via* PEEPi. These observations are strengthened by the reports of Watz and colleagues, who found a diastolic filling pattern that was more strongly associated with static hyperinflation than with airway obstruction or diffusion capacity in patients with COPD (11). In our study, the E/A ratio was above the levels observed by Watz et al., even at the highest amount of hyperinflation. We found significant increases in the E/e' ratio during dynamic hyperinflation, which is in line with studies on patients with COPD indicating significant associations between E/e' and measures of hyperinflation in body plethysmography (7, 33). Diastolic dysfunction, expressed as E/e', was again more pronounced in patients with COPD than in the participants in our study. This might be a consequence of confounding mechanisms beyond hyperinflation in COPD, which we aimed to rule out by including otherwise healthy individuals. Hence, our investigations on transmitral flow during the induction of dynamic hyperinflation in healthy subjects expands the current knowledge in the field of cardiopulmonary interaction by isolating hyperinflation from potential confounding factors in patients with COPD.

Potential mechanisms linking COPD to reduced diastolic filling and function comprise hypoxemia (18, 34), hypercapnia (35), systemic inflammation (36), arterial stiffness, pulmonary hypertension (17), and ventricular interdependence (37). Our investigation ruled out most of the abovementioned factors. Hence, potential mechanisms in our study might be limited to first, a direct connection of increased pulmonary pressure with augmented pleural stiffening. Pleural stiffness transfers to the cardiac fossa and compromises left heart dilatation due to an increased load on the ventricular wall (38). The observed increase in E/e' in the present study might indicate increased filling pressures due to mechanical constraints on the heart. Furthermore, the decrease in the E/A ratio was predominantly driven by A-velocity, which could be determined by either reduced left ventricular compliance or increased left atrial contractility (22). Second, impaired pulmonary vascular compliance (39) might reduce left ventricular preloading and, consequently, cardiac output. Interpretations of ventricular interdependence remain speculative in our study as we did not account for the end-systolic volume or stroke volume of the left ventricle during ERB. Third, the existing literature describes pulmonary hypertension as a common feature in COPD, which increases right ventricular afterload (40). We can only speculate about a potential increase in pulmonary arterial pressure during dynamic hyperinflation as the validity of echocardiographic assessments of pulmonary artery pressure *via* tricuspid regurgitation is limited during hyperinflation. We did not conduct invasive measurement of pulmonary artery pressure in our sample. Indeed, recent observations have identified pulmonary hypertension during exercise as a distinct disorder that does occur in otherwise healthy subjects (41). However, exercise pulmonary hypertension is associated with substantial dyspnea and exercise limitation, which was not observed in any of our participants.

A strength of the present study is the robust design of the randomized controlled trial. We induced dynamic hyperinflation by means of a validated method, which accurately allows the investigation of cardiopulmonary interactions in isolation from other potential mechanisms present in diseased lungs. However, it is worth mentioning that we did not measure arterial blood gases during the induction of hyperinflation. In a simulation of COPD in healthy subjects, a recent study showed that hypoxemia resulted in increased right ventricular systolic pressures (14). We cannot fully rule out a potential influence of hypoxia and increased pulmonary vascular resistance on left ventricular dysfunction during our intervention. However, Cheyne and colleagues did not find substantial changes in oxygen saturation while incrementally increasing hyperinflation with inspiratory and expiratory resistance loading (19), which makes this pathway seem negligible in our study. Ventricular interdependence as a functional impairment of the left ventricle *via* right ventricular enlargement might be a relevant effect in our study; however, this could not be evaluated due to limited access to the right ventricle by sonography during hyperinflation. As recently postulated by Farouk et al., (5) there are some concerns about assessing diastolic dysfunction in COPD using transmitral flow indices as these are strongly influenced by tachycardia, reduced preload, and ventricular septal shift. The authors showed that transmitral flow indices alone resulted in an overestimation of diastolic dysfunction compared to a recently published, more comprehensive approach (22). To address this potential confounder, we corrected the E/A ratio for a heart rate of 60/min. We excluded measurements with a relevant fusion of the E- and A-waves above a threshold of 0.2 m/s. Furthermore, we confirmed our changes in transmitral flow by tissue Doppler measurements, which revealed significant increases in the E/e' ratio indicative of diastolic dysfunction. In this study, we excluded female participants from our investigation, which might reduce the external validity of our study. On the one hand, emphysema-related hyperinflation is more pronounced in men than in women (42); on the other hand, there might be certain limitations in imaging quality with respect to echocardiography in women (43).

Finally, we did not conclusively rule out the impact of expiratory muscle activity on the amount of hyperinflation during ERB. However, during the validation of ERB we described a significant decrease in inspiratory capacity during ERB (20). In the current study, we further revealed a strong association between PEEPi and inspiratory capacity, as depicted in Supplementary Figure 1. Finally, we excluded a relevant amount of expiratory muscle activity *via* the application of a gastric balloon in one exemplary participant, illustrated in Supplementary Figure 2 and Supplementary Table 1 as previously recommended (44).

Our study indicates that hyperinflation is a determinant of impaired diastolic filling and elevated ventricular filling pressures when isolated from previously identified confounding factors. Based on our findings, the reduction of hyperinflation via bronchodilation or bronchoscopic lung volume reduction might represent a promising tool for improving diastolic dysfunction in patients with COPD.

In summary, our findings strengthen the role of dynamic hyperinflation as a determinant of left ventricular diastolic dysfunction and favor hyperinflation, in contrast to other potential mechanisms, as a treatable trait for improving left ventricular diastolic function in COPD. Future studies should test the effects of pharmacologic or endoscopic lung volume reduction on echocardiographic parameters of diastolic function and their association with clinical endpoints, such as exercise capacity or dyspnea, in patients with chronic obstructive pulmonary disease.

## Data Availability Statement

Individual participant data referred to in this article (i.e., text, tables, figures) will be made available upon reasonable request. Other available documents comprise the study protocol and statistical analysis plan. Data will be made available for researchers who provide a methodologically sound proposal. Proposals should be directed to matthias.urban@gesundheitsverbund.at (ORCID: 0000-0002-7509-4983). Researchers are required to sign a data access agreement form before gaining access to the data.

## Ethics Statement

The studies involving human participants were reviewed and approved by Ethikkommission der Stadt Wien, Thomas-Klestil-Platz 8, A-1030 Vienna, Austria. The patients/participants provided their written informed consent to participate in this study.

## Author Contributions

MU, G-CF, AM, OB, and IS: conception and design of the work. MU, G-CF, AM, and EG-S: acquisition, analysis, and interpretation of data. All authors: drafting and revising the manuscript, final approval of the version to be published, and agreement to be accountable for all aspects of the work.

## Conflict of Interest

MU received grants from Nycomed Pharma as well as speaker fees and fees for advisory boards from Astra-Zeneca, Böhringer Ingelheim, Dräger, Sanitas, and Grünenthal. IS received personal fees for lectures from Astra-Zeneca, AOP, Orphan, Böhringer-Ingelheim and Chiesi. AM and EG-S have no conflicts of interest to declare. OB received unrestricted research grants from public governmental federal institutions and and from pharma industry (Menarini, Böhringer-Ingelheim, Chiesi, GSK, Pfizer, TEVA, Astra-Zeneca Air Liquide, MSD) as a member of the Ludwig Boltzmann Institute for Lung Health for the Austrian LEAD Study. He received personal fees for lecture and as member of advisory boards from Roche, Takeda, Nycomed and Astra-Zeneca. G-CF reports speaker fees and fees for advisory boards from Boehringer Ingelheim, Menarini, Janssen-Cilag, Novartis, Insmed Germany, Getinge, Draeger, CSL Behring, Orion Pharma, Astra Zeneca, Fresenius, Chiesi, Glaxo-Smith Kline, Roche; G-CF reports educational grants from Janssen-Cilag. The remaining authors declare that the research was conducted in the absence of any commercial or financial relationships that could be construed as a potential conflict of interest.

## Abbreviations

A: transmitral doppler A-wave
COPD: chronic obstructive pulmonary disease
E: transmitral doppler E-wave
ERB: expiratory resistance breathing
e': early-diastolic tissue doppler velocity
FEV1: forced expiratory volume in 1 second
FVC: forced vital capacity
PEEPi: intrinsic positive end-expiratory pressure.
