# Supplementary material for: Effects of Dynamic Hyperinflation on Left Ventricular Diastolic Function in Healthy Subjects — A Randomized Controlled Crossover Trial
Source: Front Med (Lausanne). 2021 May 4;8:659108. doi: 10.3389/fmed.2021.659108 (PMC8129530; doi:10.3389/fmed.2021.659108)
Supplement: Supplementary file 1 [file Data_Sheet_2.docx]

Supplementary Material

**Supplementary Figure S1.** Scatter plot of the association between PEEPi (log transformed) and inspiratory capacity. (regression coefficient = -1.04; p<0.0001).


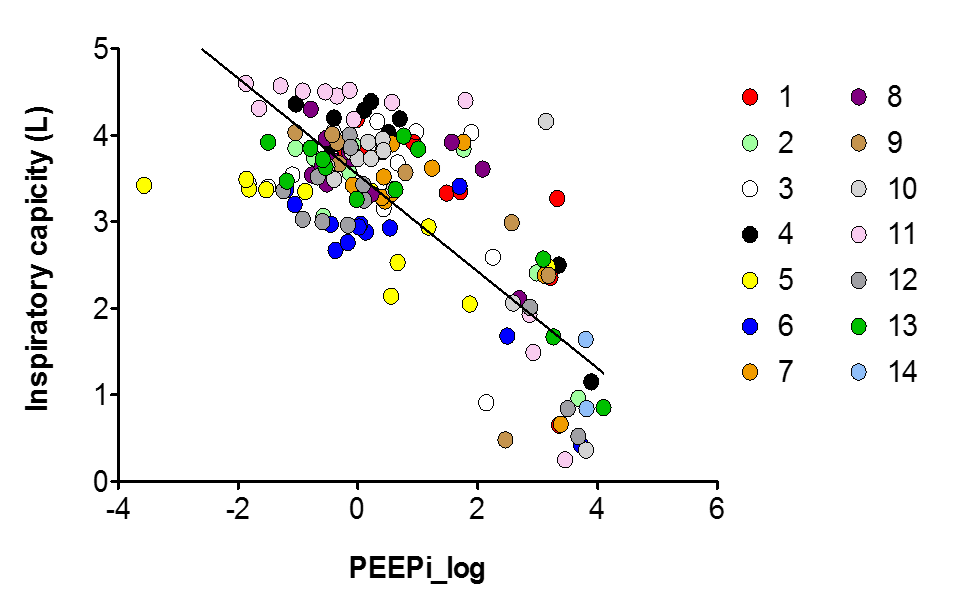


**Supplementary Figure S2.** Depiction of airway flow, esophageal pressure and gastric pressure in an exemplary participant during ERB1.


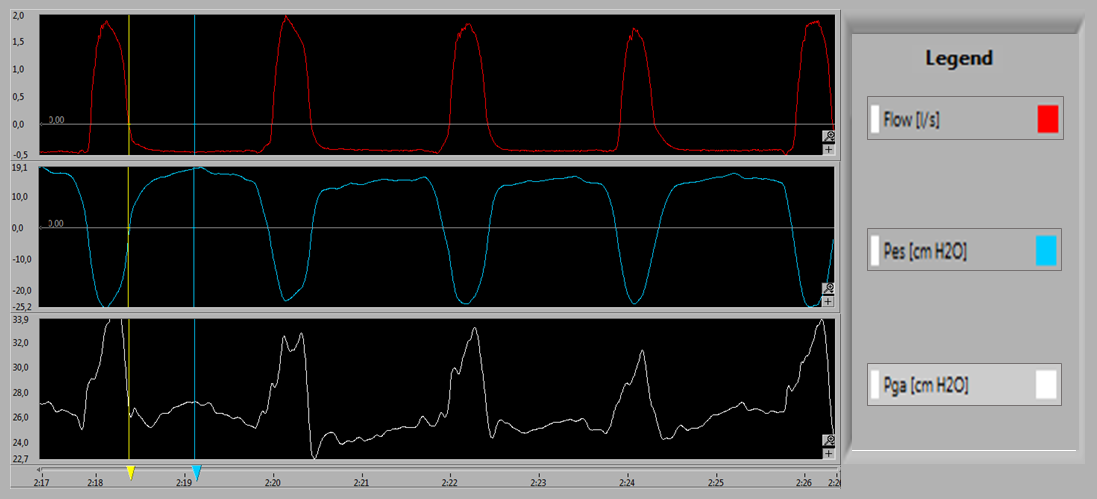


**Supplementary Table S1.** Correction of PEEPi to contribution of expiratory muscle activity in an exemplary participant during ERB1.

| **Variable** | **PEEPi (cmH2O)** | **Delta Pga (cmH2O)** | **PEEPi corrected (cmH2O)** |
| --- | --- | --- | --- |
|  | **19.79** | **1.41** | **18.38** |

Correction of PEEPi to expiratory muscle activity calculated as the initial decrease in esophageal pressure minus the increase in gastric pressure from end of expiration to peak value of the entire expiration as previously recommended (Tobin MJ. *Principles and practice of intensive care monitoring*. New York: McGraw-Hill [1998]. 1525 p.). Data are calculated as mean values of 3 consecutive respiratory cycles.
